# Combinatory Effects of Acrylamide and Deoxynivalenol on In Vitro Cell Viability and Cytochrome P450 Enzymes of Human HepaRG Cells

**DOI:** 10.3390/toxins16090389

**Published:** 2024-09-10

**Authors:** Julia Beisl, Kristina Jochum, Yi Chen, Elisabeth Varga, Doris Marko

**Affiliations:** 1 Department of Food Chemistry and Toxicology, Faculty of Chemistry, University of Vienna, 1090 Vienna, Austria; julia.beisl@outlook.com (J.B.); kristina.jochum97.kj@gmail.com (K.J.); doris.marko@univie.ac.at (D.M.); 2 German Federal Institute of Risk Assessment, Department of Pesticides Safety, Max-Dohrn-Str. 8-10, 10589 Berlin, Germany; 3 State Key Laboratory of Food Science and Technology, Nanchang University, 235 Nanjing East Road, Nanchang 330047, China; chenyi-417@163.com; 4 Unit Food Hygiene and Technology, Centre for Food Science and Veterinary Public Health, Clinical Department for Farm Animals and Food System Science, University of Veterinary Medicine, Veterinarplatz 1, 1210 Vienna, Austria

**Keywords:** food processing, hepatocytes, metabolism, mixtures, process contaminants, acrylamide, deoxynivalenol

## Abstract

Acrylamide (AA) can be formed during the thermal processing of carbohydrate-rich foods. Deoxynivalenol (DON), a mycotoxin produced by *Fusarium* spp., contaminates many cereal-based products. In addition to potential co-exposure through a mixed diet, co-occurrence of AA and DON in thermally processed cereal-based products is also likely, posing the question of combinatory toxicological effects. In the present study, the effects of AA (0.001–3 mM) and DON (0.1–30 µM) on the cytotoxicity, gene transcription, and expression of major cytochrome P450 (CYP) enzymes were investigated in differentiated human hepatic HepaRG cells. In the chosen ratios of AA–DON (10:1; 100:1), cytotoxicity was clearly driven by DON and no overadditive effects were observed. Using quantitative real-time PCR, about twofold enhanced transcript levels of *CYP1A1* were observed at low DON concentrations (0.3 and 1 µM), reflected by an increase in CYP1A activity in the ER*O*D assay. In contrast, *CYP2E1* and *CYP3A4* gene transcription decreased in a concentration-dependent manner after incubation with DON (0.01–0.3 µM). Nevertheless, confocal microscopy showed comparably constant protein levels. The present study provided no indication of an induction of *CYP2E1* as a critical step in AA bioactivation by co-occurrence with DON. Taken together, the combination of AA and DON showed no clear physiologically relevant interaction in HepaRG cells.

## 1. Introduction

Humans are generally exposed to a variety of chemicals at the same time. Food and feed, for example, may be contaminated by numerous mycotoxins simultaneously [1]. However, knowledge about the toxicity of single substances does not guarantee the correct prediction of their toxicity in a mixture [2]. Due to their generally high occurrence in foods, the mycotoxin deoxynivalenol (DON), produced by *Fusarium* spp., and acrylamide (AA), a product of the Maillard reaction, are of particular interest [3,4,5,6]. The co-occurrence of AA and DON was reported once in Latvian beer [7,8] and co-occurrence in a broad variety of heated grain-based foodstuffs, such as bread and bakery products, seems likely, as shown by [9] but has not been intensively investigated so far on a large scale. Therefore, simultaneous ingestion of the toxins within a mixed diet is highly likely.

Special attention has been drawn to AA, a low-molecular-weight, unsaturated amide, since the beginning of the 21st century, when it was first discovered in heated foods [10,11]. Since then, research on AA levels in food has increased and efforts to minimize AA in food are underway. The amount of AA formed in starch-rich foods during thermal processing depends on various factors, including the presence of precursors such as reducing sugars and asparagine and the respective process parameters [12,13]. De Borba et al. [14] showed the influence of pasta cooking on the bioaccessibility of AA. After absorption in the intestine, AA is widely distributed to various organs and tissues, for instance, the liver, where conjugation with glutathione and/or activation by cytochrome P450 (CYP) 2E1 to the highly reactive metabolite glycidamide (GA) takes place [15]. The genotoxicity of AA is mostly mediated through the metabolism to GA, which is a known mutagen, whose genotoxic effect is mediated via DNA adduct formation [4,16]. Hence, *CYP2E1* enzymes are of major importance for AA toxicity. Multiple studies investigated the effects of AA on *CYP2E1* enzymes, showing induced *CYP2E1* gene transcription and protein expression after AA treatment [17,18,19]. However, studies assessing the effects of AA on other CYP enzymes are still lacking. AA has been found to be cytotoxic in intestinal and hepatic cell lines and its anti-proliferative properties have been demonstrated in various studies [20,21,22,23].

Mycotoxins are toxic secondary metabolites of fungal origin. The mycotoxin DON, produced by *Fusarium* spp., is of major importance, and has been found to be a contaminant in cereal-based food and feed all over the world, causing severe diseases as well as substantial financial losses due to crop loss. A report by Mishra et al. [24] concerning the global occurrence of DON in food commodities indicates a potential health risk for consumers, especially infants and children. In processed foods, DON degradation was found to be as low as 2 to 6% in crackers, cookies, and bread, with iso-deoxynivalenol identified as the major degradation product [25]. Furthermore, the analysis of DON in different food groups showed DON levels of approximately 70 µg kg^−1^ in bread and rolls as well as fine bakery products [26]. Hence, though partial degradation by heating, thermally processed foods still contribute substantially to the ingestion of DON and co-contamination with substances occurring during thermal processing, such as AA, is very likely. After oral intake, DON is rapidly distributed to all tissues, including the liver [27]. Concerning the metabolic fate of DON, literature on phase I metabolism is scarce and among the various phase II metabolism pathways reported for DON in animals and humans, glucuronidation appears to be the major one [27,28]. As other trichothecenes, DON targets the ribosome and binds to its 60S subunit [29,30]. Hence, tissues undergoing permanent cell renewal and thereby requiring high rates of protein synthesis are generally more affected by DON [31]. As the human intestine is one of these tissues, in vitro studies demonstrated the cytotoxic properties of DON in several intestinal cell lines [32,33,34,35]. Once absorbed, the liver, as the major metabolizing organ, is supposedly the next potential target organ of DON. The hepatotoxic properties of DON have been less investigated in comparison to its toxic effects on the intestine. Nonetheless, cytotoxic effects were observed in different hepatic cell lines [36,37]. Since the metabolism of DON by phase I enzymes is unlikely, there is little literature on studies examining interactions in this regard. However, in vivo studies in gilts indicate that DON might influence *CYP1A1* mRNA expression [38,39].

In the present study, the effects of AA and DON, as single substances or a combination of both, on hepatic cells were investigated. The aim was to elucidate the cytotoxic properties and CYP enzyme interactions of the two substances. Different CYP-enzymes, namely *CYP1A1*, *CYP2E1*, and *CYP3A4*, were investigated due to their general importance for the metabolism of xenobiotics and pharmaceuticals and the involvement of *CYP2E1* enzymes in the metabolism of AA. Therefore, gene expression rates as well as protein expression profiles and alterations in CYP1A activity were investigated.

## 2. Results

### 2.1. Cell Viability Testing

**Figure 1:**
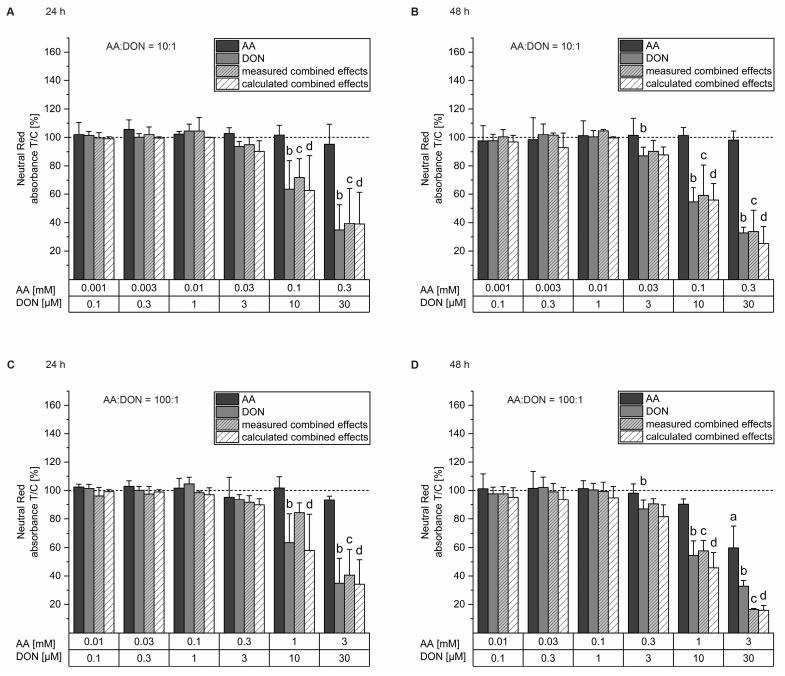
Cell viability of differentiated HepaRG cells after incubation with acrylamide (AA, dark grey), deoxynivalenol (DON, light grey), their respective combination (light grey, dashed), and the calculated combined effects (white, dashed) measured in technical triplicates by the neutral red assay after 24 h at a ratio of AA to DON of 10:1 (**A**) and 100:1 (**C**), as well as after 48 h at a ratio of AA to DON of 10:1 (**B**) and 100:1 (**D**). The results of the 4–12 biological replicates are presented as means + SD, normalized to the solvent control displayed by dashed lines. The calculated combined effects were determined by the Bliss Independence model [40,41,42]. The no-effect levels were determined by one sample Student’s *t* tests. Statistical differences with the no-effect levels were calculated by ANOVA (*p* < 0.05, Bonferroni post hoc test) and marked with the respective letters (a = AA, b = DON, c = measured combined effects, d = calculated combined effects).

To determine cell viability, the neutral red (NR) uptake assay was carried out as NR was able to be incorporated into the lysosomes of living cells. DON showed cytotoxic effects in HepaRG cells starting from 10 µM after a 24 h incubation period and decreased cell viability in a concentration-dependent manner with an estimated half-maximal inhibitory concentration (IC_50_) of 14.9 µM. At 30 µM DON, viability was diminished to 35 ± 18%. AA did not show cytotoxic effects after the 24 h incubation period, as shown in Figure 1A,C. The measured and the calculated combined effects diminished in a similar manner to DON alone. However, no significant differences were observed in their comparison. After 48 h of incubation (Figure 1B,D), AA first showed significant effects at a concentration of 3 mM leading to a cell viability of 60 ± 15%, while DON caused cytotoxic effects starting from 3 µM. At the highest applied concentration of 30 µM, DON diminished cell viability to 33 ± 4%, with an IC_50_ value of 11.6 µM. The calculated and measured combined effects decreased in a similar manner to DON alone without inducing significant differences in any condition.

### 2.2. Interferences with Gene Transcription of Selected CYP Enzymes

**Figure 2:**
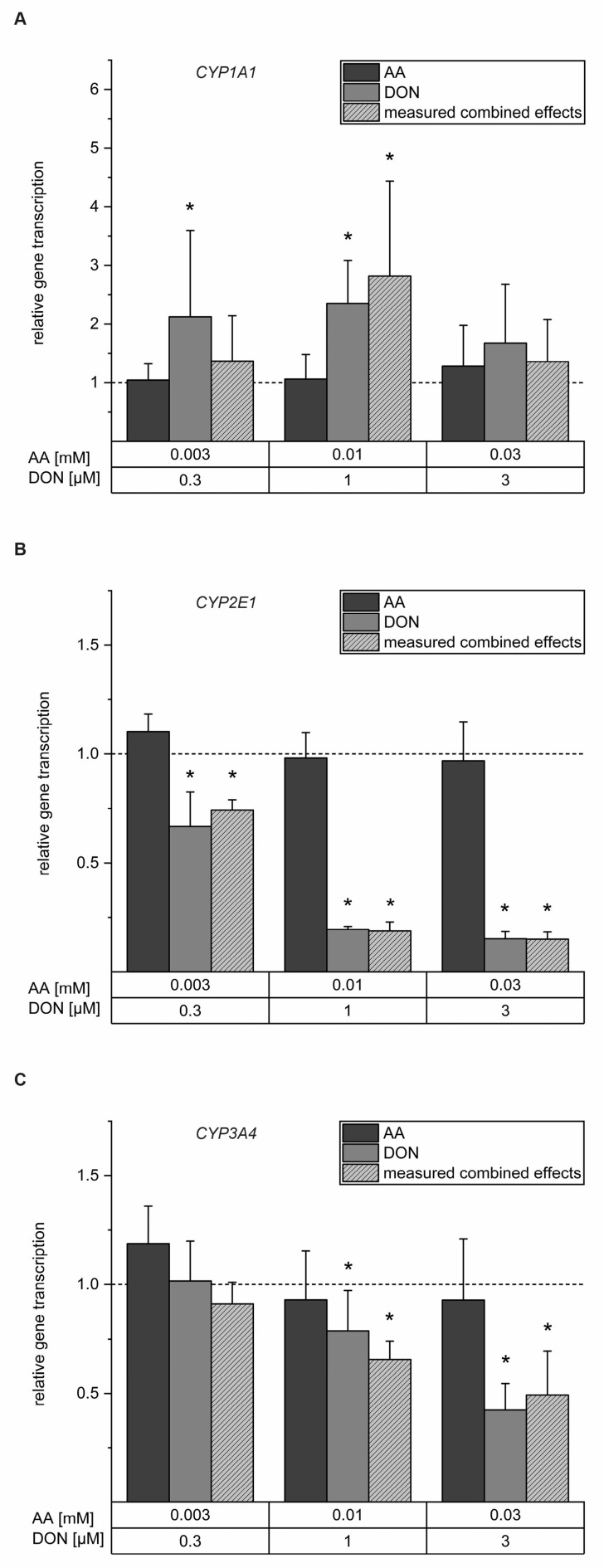
Relative quantities of the mRNA transcript levels of *CYP1A1* (**A**), *CYP2E1* (**B**), and *CYP3A4* (**C**) observed in the HepaRG cells after 24 h of incubation with acrylamide (AA, dark grey), deoxynivalenol (DON, light grey), and their respective combination (light grey, dashed) using quantitative real-time PCR. Data evaluation using the 2^−∆∆Ct^ method, according to Livak and Schmittgen [43]. All target genes were normalized to the housekeeping genes ALAS1 and HPRT1. The results are shown as means + SD of n = 5–8 independent experiments measured in technical duplicates. Statistical differences with the solvent control were calculated by the two-sample Student’s *t*-test (* *p* < 0.05). Dashed lines indicate the values obtained for the solvent controls.

*CYP2E1* enzymes catalyze the conversion of AA to GA and play a major role in human drug metabolism, together with *CYP1A1* and *CYP3A4* enzymes. Therefore, they are essential for the metabolism of various xenobiotics. Possible interferences of AA, DON, and a combination of both with transcript levels of these crucial enzymes were determined by quantitative real-time PCR (RT-qPCR). After 24 h exposure, the incubation with AA did not affect relative gene expression of *CYP1A1*, while the incubation with 0.3 and 1 µM DON induced *CYP1A1* relative gene expression to 2.13 ± 1.47 and 2.35 ± 0.73, respectively. Additionally, the combination of 0.01 mM AA and 1 µM DON increased *CYP1A1* transcript levels to 2.82 ± 1.62 (Figure 2A). The incubation with AA did not influence the mRNA expression of *CYP2E1*. However, DON and the combination of DON and AA showed a decrease in *CYP2E1* mRNA expression for all the tested concentrations (Figure 2B). The lowest tested concentration of 0.3 µM DON significantly decreased the relative gene transcription of *CYP2E1* to 0.67 ± 0.16, whereas the incubation with 1 µM and 3 µM DON decreased relative gene transcription to 0.20 ± 0.01 and 0.15 ± 0.03, respectively. A similar trend was obtained for *CYP3A4*, as shown in Figure 2C. Here, AA did not significantly influence the mRNA expression of *CYP3A4* and the lowest concentration of DON did not affect relative gene transcription (1.02 ± 0.18). In contrast, the incubation with 1 µM and 3 µM DON significantly decreased relative gene transcription to 0.79 ± 0.19 and 0.42 ± 0.12, respectively, with similar effects obtained for the combination of both substances.

### 2.3. Effects on EROD Activity

**Figure 3:**
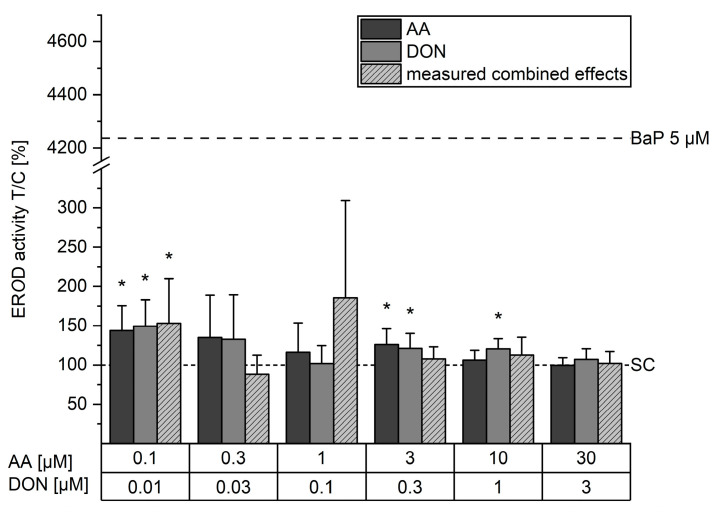
Ethoxyresorufin-*O*-deethylase activity in HepaRG cells after 24 h of incubation with acrylamide (AA, dark grey), deoxynivalenol (DON, light grey), and their respective combination (light grey, dashed) assessed with ER*O*D assay and bicinchonic acid protein assay. Results are presented as means + SD of 5–8 biological replicates measured in technical triplicates, normalized to solvent controls (SC). Statistical differences to the solvent control were determined with the one-sample Student’s *t*-test (* *p* < 0.05).

In order to clarify the obtained results for gene transcription, the well-known ethoxyresorufin-*O*-deethylases (ER*O*D) assay was carried out to determine CYP1A activity, including *CYP1A1* and CYP1A2. Benzo[a]pyrene (BaP), an inducer of *CYP1A1*, was used as a positive control and significantly increased ER*O*D activity up to 4240 ± 810% after 24 h exposure. In comparison, the incubation with 0.1 and 3 µM AA significantly induced ER*O*D activity to 144 ± 32% and 126 ± 20%, respectively. Incubation with 0.3 and 1 µM AA showed tendencies to increase ER*O*D activity but were not statistically significant. ER*O*D activity was increased significantly by 0.01, 0.3, and 1 µM DON to 150 ± 33%, 121 ± 19%, and 121 ± 13%, respectively. Additionally, the combination of 0.1 µM AA and 0.01 µM DON induced ER*O*D activity to 153 ± 57% (Figure 3).

### 2.4. Impairment of CYP2E1 and CYP3A4 Expression

**Figure 4:**
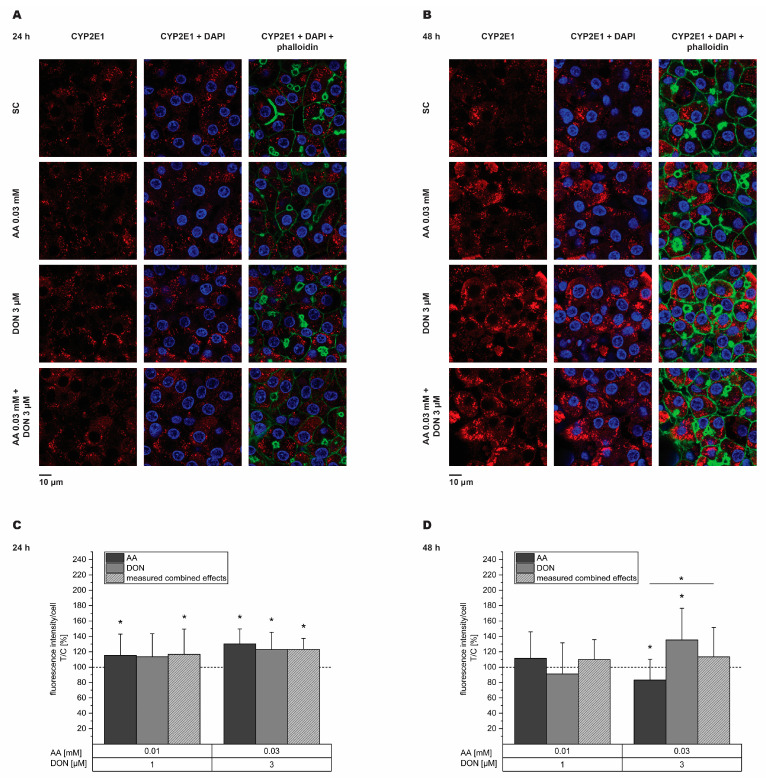
Immunofluorescence staining and subsequent quantitative analysis of *CYP2E1* enzymes after 24 h (**A**,**C**) and 48 h (**B**,**D**) incubation of HepaRG cells with acrylamide (AA, dark grey), deoxynivalenol (DON, light grey), and their respective combination (light grey, dashed). Microscopy panels (**A**,**B**) show single-channel pictures of each enzyme in first column, merge pictures with nuclei (stained with DAPI) in second column and merge pictures with nuclei and actin filaments (stained using phalloidin) in last column. Quantitative analysis is depicted in bar charts (**C**,**D**) with dashed lines displaying values of solvent controls. Results are presented as mean + SD of five biological replicates with 16–20 analyzed optical fields per condition for 24 h and four biological replicates with 13–16 analyzed optical fields per condition for 48 h. Statistical differences from the solvent control were calculated with the one-sample Student’s *t*-test (* *p* < 0.05). Statistical differences between single substances and their respective combination were calculated with the two-sample Student’s *t*-test (* *p* < 0.05).

**Figure 5:**
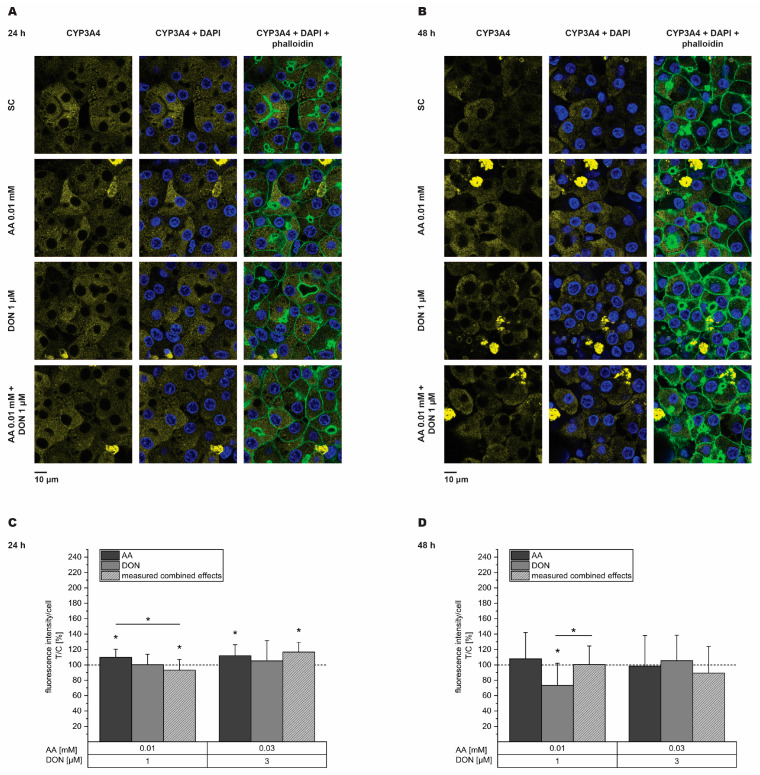
Immunofluorescence staining and subsequent quantitative analysis of *CYP3A4* enzymes after 24 h (**A**,**C**) and 48 h (**B**,**D**) incubation of HepaRG cells with acrylamide (AA, dark grey), deoxynivalenol (DON, light grey), and their respective combination (light grey, dashed). Microscopy panels (**A**,**B**) show single-channel pictures of each enzyme in first column, merge pictures with nuclei (stained with DAPI) in second column, and merge pictures with nuclei and actin filaments (stained using phalloidin) in last column. Quantitative analysis is depicted in bar charts (**C**,**D**) with dashed lines displaying the values of solvent control. Results are presented as mean + SD of five biological replicates with 17–20 analyzed optical fields per condition for 24 h and four biological replicates with 15–16 analyzed optical fields per condition for 48 h. Statistical differences to solvent control were calculated with the one-sample Student’s *t*-test (* *p* < 0.05). Statistical differences between single substance and combination were calculated with the two-sample Student’s *t*-test (* *p* < 0.05).

Immunfluorescence was used to clarify whether the observed decrease in mRNA levels of *CYP2E1* and *CYP3A4* was reflected on the protein level. As 1 µM and 3 µM DON mediated a significant decrease in mRNA expression, these two concentrations and the respective combinations with AA were further investigated. Dexamethasone (DEX; 5 µM) was tested as a positive control and significantly induced *CYP3A4* protein levels to 122 ± 32% after 48 h. After 24 h exposure to the test substances, *CYP2E1* protein expression increased for every tested concentration of the single substances and their respective combination, except 1 µM DON (Figure 4A,C). After 48 h (Figure 4B,D), *CYP2E1* significantly decreased at 0.03 mM AA and increased at 3 µM DON (83 ± 27% and 136 ± 41%, respectively). Additionally, the effect of 0.03 mM AA alone was significantly different from its combination with DON. Concerning *CYP3A4*, slight, though significant increases in protein levels were visible after 24 h for the incubation with both the AA concentrations and both the combinations of AA and DON (Figure 5A,C). After 48 h, solely the incubation with 1 µM DON showed significant effects and decreased *CYP3A4* expression to 73 ± 29% (Figure 5B,D). Comparing the effects of the single substances with their respective combination, significances were obtained for 0.01 mM AA (24 h incubation) and 1 µM DON (48 h incubation). However, both incubation periods did not reflect the effects obtained from gene transcription analysis of *CYP2E1* and *CYP3A4* after the incubation with DON.

## 3. Discussion

Cereals and cereal-based products represent a main part of global food consumption. These products are likely to be contaminated with secondary metabolites of fungal origin, including the mycotoxin DON [7]. The food processing of such grain-based products may cause the formation of AA, leading to a co-contamination of heated cereal-based products with DON and AA [7]. Considering the difficulties of predicting the toxicity of mixtures based on toxicity studies investigating single substances, this study aimed to elucidate the combinatory toxicity of AA and DON on hepatic cells [44]. As CYP enzymes play an essential role in the metabolism of AA and xenobiotics in general, the main focus was the possible interference with these enzymes.

Differentiated HepaRG cells were used as a human liver model, as after ingestion, the liver serves as the next target organ following the intestine. Additionally, the liver, as the main metabolizing organ, is of special importance for the present study, since the conversion of AA to GA takes place therein. In comparison to the well-known hepatic cell line HepG2, HepaRG cells undergo a differentiation process before their usage in toxicological assays. Hence, HepaRG cells no longer proliferate and exhibit different properties compared to HepG2 cells. The suitability of HepaRG cells for extensive in vitro toxicity studies, including CYP interactions as well as the mixture effects of a variety of substances, has been demonstrated [45,46]. Differentiated HepaRG cells exhibit CYP-dependent activities close to the levels exhibited in primary human hepatocytes and the possibility to induce or inhibit a wide variety of CYP enzymes in HepaRG cells has been shown [47,48].

In the present study, incubation with higher concentrations of all the substances significantly reduced cell viability but showed no significant differences between the measured and the calculated combined effects of AA and DON (Figure 1). Hence, we conclude that the two substances are not interacting concerning cell viability. As AA and DON act independently and there is no known interference, independent action (IA), in particular the Bliss Independence equation, was chosen as model for the calculation of combinatory effects. Although a recent report by Lasch et al. [49] suggested the use of the Chou–Talalay or benchmark dose approaches in addition to IA to ensure the correct prediction of mixture effects, their application was not possible in the present study as these approaches need dose-response curves reaching IC_50_ for the single compounds as well as for the mixtures. Analyzing the cytotoxic properties of AA, solely after exposure to 3 mM AA for 48 h was a reduction in cell viability observed. In general, data for AA cytotoxicity on differentiated HepaRG cells are still scarce, but results from a study by Le Hegarat et al. [50] using the lactate dehydrogenase assay on this cell line indicate that cytotoxic effects of AA occur at higher concentrations (10 mM) already after 24 h incubation. In contrast, multiple studies have been carried out using undifferentiated HepG2 cells, where cell viability was reduced after 24 h incubation at lower AA concentrations (~2.5 mM) than those observed for HepaRG cells [20,51]. It is assumed that the anti-proliferative properties of AA are responsible for this effect [22,23]. Regarding DON, to date, there is limited literature concerning its cytotoxic properties in differentiated HepaRG cells. However, a variety of studies conducted with HepG2 cells show cytotoxic effects after 24 h exposure to DON starting from lower concentrations (~2 µM) than those observed within this study [52,53]. As the major mode of action of DON is binding to ribosomes, DON interferes with protein synthesis [29,30]. Therefore, undifferentiated cells, such as HepG2 cells, are likely to be more affected by DON-induced toxicity than differentiated cells.

The tested concentrations and ratios in the present study were selected based on occurrence data and mimic a realistic exposure to AA and DON [4,12,24,26]. Previously, AA in vitro concentrations exceeding 6 mM were considered unrealistic [54]. Nonetheless, many in vitro studies were carried out at the highest non-cytotoxic AA concentration, e.g., 5 mM in undifferentiated Caco-2 cells, 2 mM in normal human liver cells HL7702, or 2.5 mM in HepG2 cells [17,21,55]. Regarding DON, concentrations up to approximately 30 µM were previously considered realistic [35,56]. Concerning bioavailability, in vivo studies with AA show the rapid and extensive absorption and subsequent distribution to all tissues including the liver after oral gavage [57,58,59]. Furthermore, in vitro experiments with differentiated Caco-2 cells, a well-established model for the human intestine, support the assumption that AA is rapidly absorbed and penetrates cell membranes by passive diffusion [60,61]. Similarly, the rapid absorption and distribution of DON to various tissues was demonstrated in different rodent studies [62,63,64]. In vitro experiments with differentiated Caco-2 cells underline these findings suggesting a paracellular pathway through tight junctions for intestinal DON absorbance [65,66]. Hence, under the assumption of dilution in 1 L gastric fluid the chosen test concentrations mimic a realistic hepatic exposure [35,66,67].

The enzyme responsible for the conversion of AA to its toxic metabolite GA in the liver, namely *CYP2E1*, has been demonstrated to be induced by AA [18,19]. In addition to *CYP2E1*, *CYP3A4* and *CYP1A1* play an important role in the metabolism of xenobiotics in general. In the present study, the mRNA levels of *CYP1A1*, *CYP2E1*, and *CYP3A4* were not significantly affected by 24 h incubation with 0.03 to 0.3 mM AA (Figure 2). In HepG2 cells exposed to 2.5 mM AA for 48 h, a slight induction of *CYP2E1* gene transcription was reported [17]. Hence, AA-induced *CYP2E1* gene transcription may only occur at relatively high concentrations exceeding realistic exposure levels. Concerning *CYP1A1* and *CYP3A4*, literature is scarce as their participation in the metabolism of AA to GA in humans has been described to be highly unlikely [15]. However, significantly decreased *CYP3A4* mRNA levels after 48 h exposure to 2.5 mM AA were observed in HepG2 cells [17]. In a study performed by Pyo et al. [68] investigating the combined effects of AA and ochratoxin A in HepG2 cells, no significant effects were observed for the mRNA expression of *CYP1A1* after AA treatment. However, the combination of AA and ochratoxin A induced *CYP1A1* gene transcription, emphasizing the necessity of studies investigating mixture effects. In the present study, *CYP2E1* and *CYP3A4* mRNA expression were clearly reduced following incubation with DON (Figure 2). Since DON is not known to be metabolized by phase I enzymes, few studies have investigated the effects of DON on CYP enzymes. Ivanova et al. [69] documented the interference of DON with the *CYP3A4*-mediated metabolism of enniatin B, another Fusarium mycotoxin. They suggested the possibility of DON binding close to or at a certain distance from the reaction site of the enzyme. In contrast, the present results indicate a mechanism including transcriptional interferences of DON alone. *CYP3A4* gene expression is primarily mediated through the activation of the pregnane X receptor (PXR), whose ligands include natural and synthetic steroids, as well as pharmacologically important drugs and several xenobiotics [70]. To our knowledge, interactions of DON with this receptor have not been demonstrated so far. The observed results indicate that DON induces *CYP1A1* gene transcription. As *CYP1A1* enzymes are included in the metabolism and detoxification of many xenobiotics, altered gene expression may interfere with these processes [70]. Literature on DON interactions with *CYP1A1* is still scarce. One in vitro study using the adrenocortical cell line H295R showed increased *CYP1A1* mRNA levels after DON exposure [71]. Having conducted Student’s two-sample *t*-test, no significant differences were observed between the incubation with DON and the combination of AA and DON at any concentration on *CYP1A1*, *CYP2E1*, and *CYP3A4* gene transcription. As AA did not affect *CYP1A1*, *CYP2E1*, and *CYP3A4* gene expression, the results indicated that the observed transcriptional effects were predominantly driven by DON and were not affected by co-occurrence of AA.

In order to further elucidate these effects, the CYP1A activity was investigated (Figure 3). The metabolic competence of HepaRG cells as a test system for the functional assessment of CYP1A activity and their suitability for use in the ER*O*D assay has been previously demonstrated [72,73,74]. These findings were confirmed in the present study by the high induction caused by the incubation with BaP. In comparison, the incubation with AA, DON, and their combination showed significant though marginal modulations in activity levels that might not be of physiological relevance. Hence, DON-mediated alterations of *CYP1A1* gene transcription did not manifest as altered enzyme activity. Consequently, *CYP1A1* protein expression was not further investigated.

The observed suppressive effects on *CYP2E1* and *CYP3A4* gene transcription were further studied on the protein level using immunofluorescence (IF) staining. *CYP3A4* proteins are known to be localized in the cytosol of cells, whereas *CYP2E1* proteins are associated with mitochondria and the ER [75]. Accordingly, in HepaRG cells, *CYP3A4* was found to be broadly distributed within the cells, while *CYP2E1* appeared in clustered structures (Figure 4 and Figure 5). These findings underline the successful staining of *CYP3A4* and *CYP2E1* enzymes in differentiated HepaRG cells. Additionally, *CYP2E1* and *CYP3A4* enzymes were not observed in cholangiocyte-like cells, but predominantly in hepatocyte-like clusters (Supplementary Figure S1), which accords with the results obtained by Hoekstra et al. [76], showing that hepatic functions are exclusively confined to hepatocyte-like cells. Here, a major advantage of IF analysis is the possibility to examine solely hepatocytes by selecting optical fields accordingly. Additionally, IF staining can be performed with comparatively fewer amounts of cells, as cells are cultivated in 8-well chamber slides and stained therein. Considering the quantitative analysis, significant increases and decreases, as well as significant differences between the single substance and combination, were observed between several incubation conditions, but no clear trend was visible. All around, in the investigated time window, the observed decrease in transcript levels of *CYP2E1* and *CYP3A4* were not reflected in the protein level. In a study performed by Sen et al. [17], in HepG2 cells *CYP3A4* protein levels assessed with Western blot analysis slightly decreased after AA treatment, while *CYP2E1* levels significantly increased. In contrast, Lamy et al. [77] did not detect *CYP2E1* enzyme expression in microsomes of HepG2 cells treated with AA. *CYP2E1* expression involves various mechanisms in different stages of gene expression [78]. Hence, the transcriptional inhibition of *CYP2E1* does not necessarily result in reduced protein levels, as enzyme expression, as well as protein stability, is influenced by multiple parameters. In contrast, the induction of *CYP3A4* protein expression by drug exposure is mainly achieved through transcriptional activation via PXR. In general, little is known about the influence of DON on *CYP2E1* and *CYP3A4* protein expression. However, the results of the present study raise the question of whether DON might affect the expression of CYP enzymes which are essential in drug metabolism.

## 4. Conclusions

In summary, to our knowledge, the present study is the first to show a clear reduction in *CYP2E1* and *CYP3A4* gene transcription after 24 h exposure to DON in the hepatic cell line HepaRG. Furthermore, interferences with *CYP1A1* metabolism were observed. Cell viability data strengthen the results from other studies indicating the reduced cytotoxic effects of AA and DON in differentiated cells compared to undifferentiated cells. However, within the whole investigation of CYP enzymes, no synergistic or antagonistic effects of AA and DON were observed. Nonetheless, as the present study was focusing on liver functions and a limited concentration range, interferences of AA and DON on other levels cannot be excluded. In principle, further studies are needed to investigate mixture effects, as humans are continuously exposed to mixtures of foodborne toxins and contaminants.

## 5. Materials and Methods

### 5.1. Materials

AA (A9099-25G) was purchased from Sigma-Aldrich (St. Luis, MO, USA); DON (10000318 (001101)) was purchased from Romer Labs (Tulln, Austria). Cell culture media, fetal calf serum (FCS), penicillin-streptomycin (P/S), recombinant human insulin, and Dulbecco’s phosphate-buffered saline (DPBS) were acquired from Gibco^®^ Life Technologies (Karlsruhe, Germany). Hydrocortisone 21-hemisuccinate sodium salt (HC/HS), NR powder, resorufin, 7-ethoxyresorufin (7-ER), dicumarol, BaP, and DEX were purchased from Sigma-Aldrich (St. Louis, MO, USA). DMSO, di-sodium hydrogen phosphate, EDTA, formaldehyde solution 35%, glacial acetic acid, potassium chloride, potassium di-hydrogen phosphate, sodium chloride, and Triton X-100 were obtained from Carl Roth GmbH (Karlsruhe, Germany). Trypsin was purchased from Thermo Fisher Scientific (Waltham, MA, USA); ethanol (absolute) was purchased from Chem-Lab (Zedelgem, Belgium).

### 5.2. Cell Culture

HepaRG cells were acquired from Biopredic International (Saint Grégoire, France). The hepatocarcinoma derived cells can be differentiated into two liver specific cell types, namely hepatocyte-like cells and cholangiocyte-like cells. The HepaRG cells were cultivated under humid conditions (37 °C, 5% CO_2_, 95% humidity) in a growth medium (Williams’ E medium supplemented with 10% FCS, 1% P/S, 5 µg mL^−1^ recombinant human insulin and 50 µM HC/HS). The cells were cultivated according to the supplier’s protocol. Briefly, for subcultivation, the cells were seeded in cell culture flasks at a density of 20,000 cells per cm^2^ and cultivated with a growth medium for 2 weeks. The medium was renewed every 2–3 days. Subsequently, the cells were passaged using trypsin-EDTA. For cell differentiation, the cells were seeded in cell culture flasks at a density of 25,000 cells per cm^2^ and allowed to grow in a growth medium for 2 weeks before changing to a differentiation medium (growth medium supplemented with 2% DMSO). The differentiation medium was renewed every 2–3 days for 2 weeks. Afterwards, the differentiated cells were passaged using trypsin-EDTA. The cell suspension was centrifuged for 3 min at 500× *g*, the supernatant aspirated, and the cell pellet resuspended in the differentiation medium. The cell suspension was passed through a sterile 250 µm nylon membrane to remove clots. The cells were seeded in 96-well plates, 24-well plates, or 8-well chamber plates at a density of 200,000 cells per cm^2^ for the NR experiments, RT-qPCR and CYP1A activity assays, or staining experiments, respectively. The cells were maintained in the differentiation medium for another 2 weeks to fully differentiate and used for experiments within one week.

### 5.3. Dosage Information

AA and DON stock solutions were prepared in water and stored at −80 °C and −20 °C respectively for up to 3 months. HepaRG cells were treated with 0.001–3 mM AA and 0.1–30 µM DON in differentiation medium. Differentiation medium was used as solvent control. At least 3 biological replicates were carried out for each methodology.

### 5.4. Neutral Red Uptake Assay

Cell viability was determined using the NR assay, according to Repetto et al. [79]. Briefly, the differentiated cells in the 96-well plates were treated with AA, DON, or a combination (ratios of 10:1 or 100:1) for 24 h and 48 h. Triton X-100 (0.1%) served as a positive control. NR medium was prepared by diluting a 4 mg mL^−1^ NR stock 1:100 in the HepaRG differentiation medium, incubating it overnight, and subsequently centrifuging it for 10 min at 600× *g* and filtering it through a filter paper. The incubation medium was replaced by 100 µL NR medium and incubated for 3 h. At the end of the NR incubation, the cells were washed with pre-warmed DPBS before adding 150 µL destaining solution (49.5:49.5:1 ethanol absolute, distilled water, glacial acetic acid) per well. The plate was shaken for 10 min at 500 rotations min^−1^ and 100 µL destaining solution of each well was transferred to a clear 96-well plate. Absorbance was measured immediately at 540 nm with the Cytation^TM^ 3 Cell Imaging Multi-Mode Reader (BioTek Instruments Incorporated, Winoosky, VT, USA) and was related to the solvent control set to 100%.

### 5.5. Quantitative Real-Time PCR

The influences of AA, DON, and a combination of both on the transcript rates of selected CYP enzymes were determined using RT-qPCR. Differentiated HepaRG cells were seeded in 24-well plates and treated with the test substances for 24 h. RNA extraction was performed with the Maxwell^®^ 16 LEV simplyRNA Cells Kit (Promega Corporation, Fitchburg, MA, USA) according to the manufacturer’s manual. Yield RNA concentration and purity was determined with a NanoDrop 2000c spectrometer (VWR International, Radnor, PA, USA). Reverse transcription to cDNA was performed with the QuantiTect^®^ Reverse Transcription Kit (Qiagen N.V., Venlo, The Netherlands) according to the manufacturer’s protocol. qPCR was performed with the StepOnePlus^TM^ System according to the manufacturer’s manual using QuantiTect^®^ SYBR^®^ Green Master Mix (Qiagen) and QuantiTect^®^ Primer Assays (Qiagen). The following primer assays were used: delta aminolevulinate synthase 1 (ALAS1, QT00073122), cytochrome P450 1A1 (*CYP1A1*, QT00012341), cytochrome P450 3A4 (*CYP3A4*, QT00024969), cytochrome P450 2E1 (*CYP2E1*, QT01669962), and hypoxanthine phosphoribosyltransferase 1 (HPRT1, QT00059066). The following PCR protocol was used for amplification: enzyme activation at 95 °C for 15 min, 40 cycles of 15 s at 94 °C, 30 s at 55 °C, and 30 s at 72 °C, followed by melting curve analysis: 15 s at 95 °C, 1 min at 60 °C, in 0.5 °C steps for 15 s to 94 °C. The data were analyzed with the StepOnePlus^®^ v2.1 software, normalized to the mean of two endogenous control genes (ALAS1 and HPRT1), and quantified with the 2^−∆∆Ct^ method [43]. Primer efficiency was tested beforehand according to Schmittgen and Livak [80].

### 5.6. CYP1A Activity

CYP1A activity was determined utilizing the CYP1A-dependent metabolism of ER*O*D by formation of fluorescent resorufin. The differentiated HepaRG cells in 24-well plates were treated with AA, DON, and a combination of both for 24 h. BaP (5 µM) was used as a positive control. At the end of the incubation period, the incubation medium was replaced by 400 µL colourless Williams’ E containing 10 µM 7-ER and 10 µM dicumarol (ER*O*D medium) and incubated for 30 min. At the end of the ER*O*D incubation time, the supernatant of each well was transferred into pre-cooled tubes under the exclusion of light. An aliquot (75 µL) of each supernatant medium was transferred into a black microtiter plate already containing 200 µL of 99.8% ethanol. The fluorescence was measured immediately in the Cytation^TM^ 3 Cell Imaging Multi-Mode Reader (BioTek Instruments Incorporated, Winoosky, VT, USA) at 535 nm excitation and 595 nm emission. The resorufin concentration was determined by the relation to a standard curve ranging from 0 to 25 nM.

After the ER*O*D incubation, the cells were washed with the colourless Williams’ E medium, the medium was aspirated, and the 24-well plate was immediately placed at −80 °C. Cell lysis was performed by at least three thawing and freezing cycles. The protein content was assessed with bicinchonic acid Protein Assay (Merck KGaA, Darmstadt, Germany) according to the manufacturer’s protocol. The determined CYP1A activity was normalized to the protein content of each sample and related to the solvent control set to 100%.

### 5.7. Immunofluorescence Staining

Indirect IF staining with subsequent microscopic analysis was performed as previously described in order to study the expression of *CYP3A4* and *CYP2E1* enzymes [32,81]. HepaRG cells were seeded in 8-well chamber slides (ibiTreat, Ibidi GmbH, Gräfelfing, Germany) and incubated with the test substances for 24 h or 48 h. DEX (10 µM) served as a positive control. At the end of the incubation period, cells were washed with PBS and a fixation took place by the addition of pre-warmed 3.7% formaldehyde in PBS for 15 min. The cells were washed twice with PBS and permeabilization took place by the addition of 0.2% Triton X-100 in PBS for 10 min. The cells were washed with PBS again and blocking solution, consisting of 1% donkey serum (Sigma-Aldrich, St. Louis, MO, USA) in PBS, was added. After 1 h, the blocking solution was replaced by primary antibodies diluted in 0.5% donkey serum in PBS (*CYP3A4* monoclonal Antibody, MA-17064, Thermo Fisher Scientific Inc., Waltham, MA, USA, 1:300; Anti-Cytochrome P450 2E1 antibody, ab28146, Abcam plc. Cambridge, UK, 1:500). The cells were treated with primary antibodies for 2 h. Afterwards, the cells were washed 3 times for 10 min with wash buffer (0.05% Triton X-100 in PBS) and then twice with PBS. Secondary antibodies diluted in 0.5% donkey serum in PBS (Alexa Fluor^TM^ 647 donkey anti-mouse IgG (H + L), Thermo Fisher Scientific Inc., 1:1000; Alexa Fluor^TM^ 568 donkey anti-rabbit IgG (H + L), 1:1000, Thermo Fisher Scientific Inc.; Oregon Green^®^ 488 phalloidin, Thermo Fisher Scientific Inc., 1:500) were added and incubated under the exclusion of light for 1.5 h. The same washing procedure as before was carried out. Post-staining fixation was performed by treating the cells with 3.7% formaldehyde in PBS for 10 min followed by 100 mM glycine in PBS, in order to mask the reactive sites. Per well, 3 drops Roti^®^-Mount FluorCare DAPI mounting media (Carl Roth GmbH + Co. KG, Karlsruhe, Germany) were added. The chamber slides were stored at 4 °C, protected from light, until the examination with an LSM Zeiss 710 microscope (ZEISS, Oberkochen, Germany) equipped with an ELYRA PS.1 system (ZEISS, Oberkochen, Germany), an AndoriXon 897 (EMCCD) camera (Oxford Instruments plc, Abingdon, UK), and a Plan Apochromat 100× (1.46 NA) objective (ZEISS, Oberkochen, Germany). Image analysis was performed with ZEN 3.0 (2012) software. For each incubation condition, 4 optical fields per biological replicate were randomly chosen, making sure that the optical field only consisted of hepatocyte-like cells, resulting in the analysis of at least 16 optical fields per condition in total. The sum of fluorescence intensity of each optical field was taken and related to the number of nuclei. The mean value as the fluorescence intensity per nuclei (FI nuclei^−1^) for all 4 optical fields was calculated for the SC. Each FI nuclei^−1^ value of each optical field and condition was related to the mean FI nuclei^−1^ of the solvent control.

### 5.8. Statistical Analysis

OriginPro 2021 was used for all statistical analysis and Microsoft Excel 2013 and 2016 for all mathematical analysis. Testing for normal distribution of results was carried out with Shapiro–Wilk normality test. Nalimov outliner test was applied to all results. Where possible, namely for cell viability results, combined effects (f_combined_) were calculated from effects of single substances (f_AA_ and f_DON_) with Bliss Independence equation f_combined_ = f_AA_ + f_DON_ − f_AA_ × f_DON_ with f representing cytotoxic effect, calculated as 1 minus cell viability relative to solvent control [40,41,42]. Student’s two-sample *t*-test was performed to compare calculated combined effects to measured combined effects and to compare effect of a single substance with effect of respective combination. One-way ANOVA followed by Bonferroni post hoc test was performed to compare effects of one substance at different concentrations. *p* < 0.05 was used in all statistical analyses.

## Data Availability

Dataset available on request from the authors.

## Supplementary Materials

The following supporting information can be downloaded at: https://www.mdpi.com/article/10.3390/toxins16090389/s1; Figure S1: Microscopic observation (10×) of differentiated HepaRG cells with a confocal microscope (A) and with a bright-field microscope (10×) (B).

## References

[1] Smith M.-C., Madec S., Coton E., Hymery N. (2016). Natural Co-Occurrence of Mycotoxins in Foods and Feeds and their in vitro Combined Toxicological Effects. Toxins.

[2] Heys K.A., Shore R.F., Pereira M.G., Jones K.C., Martin F.L. (2016). Risk assessment of environmental mixture effects. RSC Adv..

[3] WHO|JECFA Safety Evaluation of Certain Contaminants in Food—Acrylamide. WHO Food Additives Series 63, FAO JECFA Monographs 8..

[4] (2015). Scientific Opinion on acrylamide in food. EFSA J..

[5] Wang Z., Wu Q., Kuča K., Dohnal V., Tian Z. (2014). Deoxynivalenol: Signaling pathways and human exposure risk assessment—An update. Arch. Toxicol..

[6] Munkvold G.P. (2017). *Fusarium* Species and Their Associated Mycotoxins. Methods Mol. Biol..

[7] Thielecke F., Nugent A.P. (2018). Contaminants in Grain-A Major Risk for Whole Grain Safety?. Nutrients.

[8] Bogdanova E., Rozentale I., Pugajeva I., Emecheta E.E., Bartkevics V. (2018). Occurrence and risk assessment of myco-toxins, acrylamide, and furan in Latvian beer. Food Addit. Contam. Part B Surveill..

[9] Balbo C., Woźniak Ł. (2022). Dietary exposure and risk characterisation of multiple chemical contaminants in rye-wheat bread marketed in Poland. EFSA J..

[10] Tareke E., Rydberg P., Karlsson P., Eriksson S., Törnqvist M. (2002). Analysis of acrylamide, a carcinogen formed in heated foodstuffs. J. Agric. Food Chem..

[11] Koszucka A., Nowak A. (2019). Thermal processing food-related toxicants: A review. Crit. Rev. Food Sci. Nutr..

[12] Semla M., Goc Z., Martiniaková M., Omelka R., Formicki G. (2017). Acrylamide: A common food toxin related to physiological functions and health. Physiol. Res..

[13] Taeymans D., Wood J., Ashby P., Blank I., Studer A., Stadler R.H., Gondé P., van Eijck P., Lalljie S., Lingnert H. (2004). A review of acrylamide: An industry perspective on research, analysis, formation, and control. Crit. Rev. Food Sci. Nutr..

[14] De Borba V.S., Lemos A.C., Cerqueira M.B.R., Badiale-Furlong E. (2023). Pasta cooking influence on in vitro bioaccessibility of type B trichothecenes, acrylamide and hydroxymethylfurfural. Food Res. Int..

[15] Kraus D., Rokitta D., Fuhr U., Tomalik-Scharte D. (2013). The role of human cytochrome P450 enzymes in metabolism of acrylamide in vitro. Toxicol. Mech. Methods.

[16] Ghanayem B.I., McDaniel L.P., Churchwell M.I., Twaddle N.C., Snyder R., Fennell T.R., Doerge D.R. (2005). Role of CYP2E1 in the epoxidation of acrylamide to glycidamide and formation of DNA and hemoglobin adducts. Toxicol. Sci..

[17] Sen A., Ozgun O., Arinç E., Arslan S. (2012). Diverse action of acrylamide on cytochrome P450 and glutathione S-transferase isozyme activities, mRNA levels and protein levels in human hepatocarcinoma cells. Cell Biol. Toxicol..

[18] Mei N., Guo L., Tseng J., Dial S.L., Liao W., Manjanatha M.G. (2008). Gene expression changes associated with xenobiotic metabolism pathways in mice exposed to acrylamide. Environ. Mol. Mutagen..

[19] Nixon B.J., Katen A.L., Stanger S.J., Schjenken J.E., Nixon B., Roman S.D. (2014). Mouse spermatocytes express CYP2E1 and respond to acrylamide exposure. PLoS ONE.

[20] Cao J., Liu Y., Jia L., Jiang L.-P., Geng C.-Y., Yao X.-F., Kong Y., Jiang B.-N., Zhong L.-F. (2008). Curcumin attenuates acrylamide-induced cytotoxicity and genotoxicity in HepG2 cells by ROS scavenging. J. Agric. Food Chem..

[21] Chen W., Feng L., Shen Y., Su H., Li Y., Zhuang J., Zhang L., Zheng X. (2013). Myricitrin inhibits acrylamide-mediated cytotoxicity in human Caco-2 cells by preventing oxidative stress. Biomed. Res. Int..

[22] Kacar S., Vejselova D., Kutlu H.M., Sahinturk V. (2018). Acrylamide-derived cytotoxic, anti-proliferative, and apoptotic effects on A549 cells. Hum. Exp. Toxicol..

[23] Chen J.-H., Tsou T.-C., Chiu I.-M., Chou C.-C. (2010). Proliferation inhibition, DNA damage, and cell-cycle arrest of human astrocytoma cells after acrylamide exposure. Chem. Res. Toxicol..

[24] Mishra S., Srivastava S., Dewangan J., Divakar A., Kumar Rath S. (2020). Global occurrence of deoxynivalenol in food commodities and exposure risk assessment in humans in the last decade: A survey. Crit. Rev. Food Sci. Nutr..

[25] Stadler D., Lambertini F., Woelflingseder L., Schwartz-Zimmermann H., Marko D., Suman M., Berthiller F., Krska R. (2019). The Influence of Processing Parameters on the Mitigation of Deoxynivalenol during Industrial Baking. Toxins.

[26] European Food Safety Authority (2013). Deoxynivalenol in food and feed: Occurrence and exposure. EFSA J..

[27] Payros D., Alassane-Kpembi I., Pierron A., Loiseau N., Pinton P., Oswald I.P. (2016). Toxicology of deoxynivalenol and its acetylated and modified forms. Arch. Toxicol..

[28] Warth B., Sulyok M., Berthiller F., Schuhmacher R., Krska R. (2013). New insights into the human metabolism of the Fusarium mycotoxins deoxynivalenol and zearalenone. Toxicol. Lett..

[29] EFSA CONTAM Panel (EFSA Panel on Contaminants in the Food Chain), Knutsen, H (2017). K.; Alexander, J.; Barregård, L.; Bignami, M.; Brüschweiler, B.; Ceccatelli, S.; Cottrill, B.; Dinovi, M.; Grasl-Kraupp, B.; et al. Scientific Opinion on the risks to human and animal health related to the presence of deoxynivalenol and its acetylated and modified forms in food and feed. EFSA J..

[30] Ueno Y. (1977). Mode of action of trichothecenes. Ann. Nutr. Aliment..

[31] Bony S., Carcelen M., Olivier L., Devaux A. (2006). Genotoxicity assessment of deoxynivalenol in the Caco-2 cell line model using the Comet assay. Toxicol. Lett..

[32] Del Favero G., Woelflingseder L., Braun D., Puntscher H., Kütt M.-L., Dellafiora L., Warth B., Pahlke G., Dall’Asta C., Adam G. (2018). Response of intestinal HT-29 cells to the trichothecene mycotoxin deoxynivalenol and its sulfated conjugates. Toxicol. Lett..

[33] Instanes C., Hetland G. (2004). Deoxynivalenol (DON) is toxic to human colonic, lung and monocytic cell lines, but does not increase the IgE response in a mouse model for allergy. Toxicology.

[34] Maresca M., Mahfoud R., Garmy N., Fantini J. (2002). The mycotoxin deoxynivalenol affects nutrient absorption in human intestinal epithelial cells. J. Nutr..

[35] Beisl J., Pahlke G., Abeln H., Ehling-Schulz M., Del Favero G., Varga E., Warth B., Sulyok M., Abia W., Ezekiel C.N. (2020). Combinatory effects of cereulide and deoxynivalenol on in vitro cell viability and inflammation of human Caco-2 cells. Arch. Toxicol..

[36] Peng Z., Chen L., Nüssler A.K., Liu L., Yang W. (2017). Current sights for mechanisms of deoxynivalenol-induced hepatotoxicity and prospective views for future scientific research: A mini review. J. Appl. Toxicol..

[37] Königs M., Schwerdt G., Gekle M., Humpf H.-U. (2008). Effects of the mycotoxin deoxynivalenol on human primary hepatocytes. Mol. Nutr. Food Res..

[38] Reddy K.E., Lee W., Jeong J.Y., Lee Y., Lee H.-J., Kim M.S., Kim D.-W., Yu D., Cho A., Oh Y.K. (2018). Effects of deoxynivalenol- and zearalenone-contaminated feed on the gene expression profiles in the kidneys of piglets. Asian-Australas. J. Anim. Sci..

[39] Gajęcka M., Dąbrowski M., Otrocka-Domagała I., Brzuzan P., Rykaczewska A., Cieplińska K., Barasińska M., Gajęcki M.T., Zielonka Ł. (2020). Correlations between exposure to deoxynivalenol and zearalenone and the immunohistochemical expression of estrogen receptors in the intestinal epithelium and the mRNA expression of selected colonic enzymes in pre-pubertal gilts. Toxicon.

[40] Chou T.-C. (2006). Theoretical basis, experimental design, and computerized simulation of synergism and antagonism in drug combination studies. Pharmacol. Rev..

[41] Bliss C.I. (1939). The Toxicity of Poisons Applied Jointly. Ann. Appl. Biol..

[42] Webb J.L. (1963). Effect of more than one inhibitor. Enzym. Metab. Inhib..

[43] Livak K.J., Schmittgen T.D. (2001). Analysis of relative gene expression data using real-time quantitative PCR and the 2(-Delta Delta C(T)) Method. Methods.

[44] Klarić M.S., Rašić D., Peraica M. (2013). Deleterious effects of mycotoxin combinations involving ochratoxin A. Toxins.

[45] Knebel C., Neeb J., Zahn E., Schmidt F., Carazo A., Holas O., Pavek P., Püschel G.P., Zanger U.M., Süssmuth R. (2018). Unexpected Effects of Propiconazole, Tebuconazole, and Their Mixture on the Receptors CAR and PXR in Human Liver Cells. Toxicol. Sci..

[46] Knebel C., Kebben J., Eberini I., Palazzolo L., Hammer H.S., Süssmuth R.D., Heise T., Hessel-Pras S., Lampen A., Braeuning A. (2018). Propiconazole is an activator of AHR and causes concentration additive effects with an established AHR ligand. Arch. Toxicol..

[47] Hartman G.D., Kuduk S.D., Espiritu C., Lam A.M. (2020). P450s under Restriction (PURE) Screen Using HepaRG and Primary Human Hepatocytes for Discovery of Novel HBV Antivirals. ACS Med. Chem. Lett..

[48] Andersson T.B., Kanebratt K.P., Kenna J.G. (2012). The HepaRG cell line: A unique in vitro tool for understanding drug metabolism and toxicology in human. Expert Opin. Drug Metab. Toxicol..

[49] Lasch A., Lichtenstein D., Marx-Stoelting P., Braeuning A., Alarcan J. (2020). Mixture effects of chemicals: The difficulty to choose appropriate mathematical models for appropriate conclusions. Environ. Pollut..

[50] Le Hegarat L., Dumont J., Josse R., Huet S., Lanceleur R., Mourot A., Poul J.-M., Guguen-Guillouzo C., Guillouzo A., Fessard V. (2010). Assessment of the genotoxic potential of indirect chemical mutagens in HepaRG cells by the comet and the cytokinesis-block micronucleus assays. Mutagenesis.

[51] Chen X., Murdoch R., Shafer D.J., Ajuwon K.M., Applegate T.J. (2016). Cytotoxicity of various chemicals and mycotoxins in fresh primary duck embryonic fibroblasts: A comparison to HepG2 cells. J. Appl. Toxicol..

[52] Juan-García A., Taroncher M., Font G., Ruiz M.-J. (2018). Micronucleus induction and cell cycle alterations produced by deoxynivalenol and its acetylated derivatives in individual and combined exposure on HepG2 cells. Food Chem. Toxicol..

[53] Juan-García A., Juan C., Tolosa J., Ruiz M.J. (2019). Effects of deoxynivalenol, 3-acetyl-deoxynivalenol and 15-acetyl-deoxynivalenol on parameters associated with oxidative stress in HepG2 cells. Mycotoxin Res..

[54] Eisenbrand G. (2020). Revisiting the evidence for genotoxicity of acrylamide (AA), key to risk assessment of dietary AA exposure. Arch. Toxicol..

[55] Hou L., Liu S., Zhao C., Fan L., Hu H., Yin S. (2021). The combination of T-2 toxin and acrylamide synergistically induces hepatotoxicity and nephrotoxicity via the activation of oxidative stress and the mitochondrial pathway. Toxicon.

[56] Van de Walle J., Romier B., Larondelle Y., Schneider Y.-J. (2008). Influence of deoxynivalenol on NF-kappaB activation and IL-8 secretion in human intestinal Caco-2 cells. Toxicol. Lett..

[57] Kim T.H., Shin S., Kim K.B., Seo W.S., Shin J.C., Choi J.H., Weon K.-Y., Joo S.H., Jeong S.W., Shin B.S. (2015). Determination of acrylamide and glycidamide in various biological matrices by liquid chromatography-tandem mass spectrometry and its application to a pharmacokinetic study. Talanta.

[58] Doerge D.R., Young J.F., McDaniel L.P., Twaddle N.C., Churchwell M.I. (2005). Toxicokinetics of acrylamide and glycidamide in Fischer 344 rats. Toxicol. Appl. Pharmacol..

[59] Doerge D.R., Young J.F., McDaniel L.P., Twaddle N.C., Churchwell M.I. (2005). Toxicokinetics of acrylamide and glycidamide in B6C3F1 mice. Toxicol. Appl. Pharmacol..

[60] Zödl B., Schmid D., Wassler G., Gundacker C., Leibetseder V., Thalhammer T., Ekmekcioglu C. (2007). Intestinal transport and metabolism of acrylamide. Toxicology.

[61] Schabacker J., Schwend T., Wink M. (2004). Reduction of acrylamide uptake by dietary proteins in a Caco-2 gut model. J. Agric. Food Chem..

[62] Meky F.A., Turner P.C., Ashcroft A.E., Miller J.D., Qiao Y.-L., Roth M.J., Wild C.P. (2003). Development of a urinary biomarker of human exposure to deoxynivalenol. Food Chem. Toxicol..

[63] Pestka J.J., Islam Z., Amuzie C.J. (2008). Immunochemical assessment of deoxynivalenol tissue distribution following oral exposure in the mouse. Toxicol. Lett..

[64] Wan D., Huang L., Pan Y., Wu Q., Chen D., Tao Y., Wang X., Liu Z., Li J., Wang L. (2014). Metabolism, distribution, and excretion of deoxynivalenol with combined techniques of radiotracing, high-performance liquid chromatography ion trap time-of-flight mass spectrometry, and online radiometric detection. J. Agric. Food Chem..

[65] Beisl J., Varga E., Braun D., Warth B., Ehling-Schulz M., Del Favero G., Marko D. (2021). Assessing Mixture Effects of Cereulide and Deoxynivalenol on Intestinal Barrier Integrity and Uptake in Differentiated Human Caco-2 Cells. Toxins.

[66] Sergent T., Parys M., Garsou S., Pussemier L., Schneider Y.-J., Larondelle Y. (2006). Deoxynivalenol transport across human intestinal Caco-2 cells and its effects on cellular metabolism at realistic intestinal concentrations. Toxicol. Lett..

[67] Ling K.-H., Wan M.L.Y., El-Nezami H., Wang M. (2016). Protective Capacity of Resveratrol, a Natural Polyphenolic Compound, against Deoxynivalenol-Induced Intestinal Barrier Dysfunction and Bacterial Translocation. Chem. Res. Toxicol..

[68] Pyo M.C., Shin H.S., Jeon G.Y., Lee K.-W. (2020). Synergistic Interaction of Ochratoxin A and Acrylamide Toxins in Human Kidney and Liver Cells. Biol. Pharm. Bull..

[69] Ivanova L., Denisov I.G., Grinkova Y.V., Sligar S.G., Fæste C.K. (2019). Biotransformation of the Mycotoxin Enniatin B1 by CYP P450 3A4 and Potential for Drug-Drug Interactions. Metabolites.

[70] Manikandan P., Nagini S. (2018). Cytochrome P450 Structure, Function and Clinical Significance: A Review. Curr. Drug Targets.

[71] Ndossi D.G., Frizzell C., Tremoen N.H., Fæste C.K., Verhaegen S., Dahl E., Eriksen G.S., Sørlie M., Connolly L., Ropstad E. (2012). An in vitro investigation of endocrine disrupting effects of trichothecenes deoxynivalenol (DON), T-2 and HT-2 toxins. Toxicol. Lett..

[72] Mann A., Pelz T., Rennert K., Mosig A., Decker M., Lupp A. (2017). Evaluation of HepaRG cells for the assessment of indirect drug-induced hepatotoxicity using INH as a model substance. Hum. Cell.

[73] Bernasconi C., Pelkonen O., Andersson T.B., Strickland J., Wilk-Zasadna I., Asturiol D., Cole T., Liska R., Worth A., Müller-Vieira U. (2019). Validation of in vitro methods for human cytochrome P450 enzyme induction: Outcome of a multi-laboratory study. Toxicol. In Vitro.

[74] Abass K., Lämsä V., Reponen P., Küblbeck J., Honkakoski P., Mattila S., Pelkonen O., Hakkola J. (2012). Characteriza-tion of human cytochrome P450 induction by pesticides. Toxicology.

[75] Pontén F., Jirström K., Uhlen M. (2008). The Human Protein Atlas—A tool for pathology. J. Pathol..

[76] Hoekstra R., Nibourg G.A.A., van der Hoeven T.V., Ackermans M.T., Hakvoort T.B.M., van Gulik T.M., Lamers W.H., Elferink R.P.O., Chamuleau R.A.F.M. (2011). The HepaRG cell line is suitable for bioartificial liver application. Int. J. Biochem. Cell Biol..

[77] Lamy E., Völkel Y., Roos P.H., Kassie F., Mersch-Sundermann V. (2008). Ethanol enhanced the genotoxicity of acrylamide in human, metabolically competent HepG2 cells by CYP2E1 induction and glutathione depletion. Int. J. Hyg. Environ. Health.

[78] Martignoni M., Groothuis G.M.M., de Kanter R. (2006). Species differences between mouse, rat, dog, monkey and human CYP-mediated drug metabolism, inhibition and induction. Expert Opin. Drug Metab. Toxicol..

[79] Repetto G., Del Peso A., Zurita J.L. (2008). Neutral red uptake assay for the estimation of cell viability/cytotoxicity. Nat. Protoc..

[80] Schmittgen T.D., Livak K.J. (2008). Analyzing real-time PCR data by the comparative C(T) method. Nat. Protoc..

[81] Beisl J., Pahlke G., Ehling-Schulz M., Del Favero G., Marko D. (2022). Cereulide and Deoxynivalenol Increase LC3 Protein Levels in HepG2 Liver Cells. Toxins.

