# Supplementary figures and images for: Combinatory Effects of Acrylamide and Deoxynivalenol on In Vitro Cell Viability and Cytochrome P450 Enzymes of Human HepaRG Cells
Source: Toxins (Basel). 2024 Sep 10;16(9):389. doi: 10.3390/toxins16090389 (PMC11436166; doi:10.3390/toxins16090389)

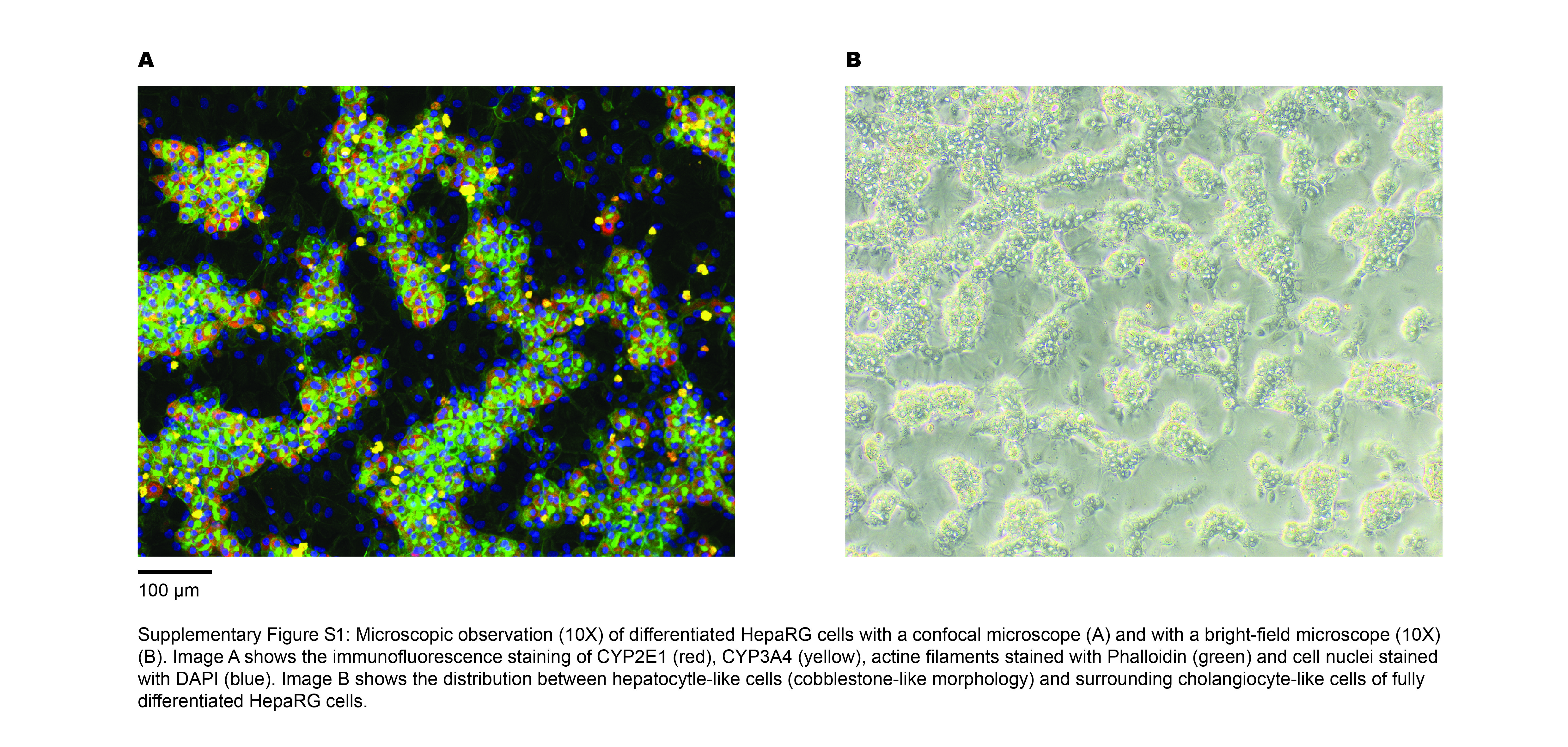

Supplement: Supplementary file 1 [file toxins-16-00389-s001.zip › toxins-3168506-supplementary.jpg]
